# HBV preS deletion mapping using deep sequencing demonstrates a unique association with viral markers

**DOI:** 10.1371/journal.pone.0212559

**Published:** 2019-02-22

**Authors:** Yuichiro Suzuki, Shinya Maekawa, Nobutoshi Komatsu, Mitsuaki Sato, Akihisa Tatsumi, Mika Miura, Shuya Matsuda, Masaru Muraoka, Natsuko Nakakuki, Fumitake Amemiya, Shinichi Takano, Mitsuharu Fukasawa, Yasuhiro Nakayama, Tatsuya Yamaguchi, Taisuke Inoue, Tadashi Sato, Minoru Sakamoto, Atsuya Yamashita, Kohji Moriishi, Nobuyuki Enomoto

**Affiliations:** 1 First Department of Internal Medicine, Faculty of Medicine, University of Yamanashi, Chuo, Yamanashi, Japan; 2 Department of Microbiology, University of Yamanashi, Chuo, Yamanashi, Japan; University of Cincinnati College of Medicine, UNITED STATES

## Abstract

**Aim:**

Deletions are observed frequently in the preS1/S2 region of hepatitis B virus (HBV) genome, in association with liver disease advancement. However, the most significant preS1/S2 region and its influences on viral markers are unclear.

**Methods:**

The preS1/S2 HBV regions of 90 patients without antiviral therapy were subjected to deep sequencing and deleted regions influencing viral markers were investigated.

**Results:**

From the deletion frequency analysis in each patient, deletions were observed most frequently in the preS2 codon 132–141 region. When the patients were divided into three groups (0–0.1%: n = 27, 0.1%-10%: n = 34, 10–100%: n = 29), based on the deletion frequency, FIB-4 (p < 0.01), HBV DNA (p < 0.01), HBcrAg (p < 0.01) and preS1/S2 start codon mutations (p < 0.01, both) were significantly associated with the deletion. When clinical and viral markers were investigated by multivariate analysis for their association with the deletion, FIB-4 (p < 0.05), HBcrAg (p < 0.05), and preS1 start codon mutation (p < 0.01) were extracted as independent variables. When the influence of the preS codon 132-141deletions on HBsAg and HBcrAg, relative to HBV DNA, was investigated, the HBsAg/HBV DNA ratio was lower (0–10% vs. 10%-100%, p<0.05), while the HBcrAg/HBV DNA rati o was higher (0–0.1% vs. 10%-100%, p<0.05) in the presence of the preS codon 132-141deletions.

**Conclusion:**

The preS codon.132-141 deletions have a significant influence on the clinical characteristics and viral markers, even when present as a minor population. Importantly, the preS codon 132–141 deletions have a clear influence on the viral life cycle and pathogenesis.

## Introduction

Hepatitis B virus (HBV) chronically infects more than 257 million people worldwide and increases the risk of these individuals developing liver cirrhosis, hepatic decompensation and hepatocellular carcinoma (HCC) over the long course of the disease [1]. Recent advances in the development of nucleoside and nucleotide analogues (NAs) have made it possible to decrease hepatitis activity and to suppress serum hepatitis B virus DNA (HBV DNA) dramatically. However, it is also acknowledged that HCC may develop in a substantial number of patients, even after the introduction of these NAs, while prediction of those patients who will develop liver disease after NA introduction is difficult. Consequently, appropriate biomarkers that predict disease development are needed urgently. HBV markers, such as genomic sequences and viral proteins, are candidates for such biomarkers but the precise roles of these viral markers for disease advancement are not fully understood.

The preS region of the HBV genome comprises preS1 and preS2 and it is known that various mutations are often found there, along with liver disease advancement, and that deletions are the most frequent [2]. These mutations are considered to occur as a result of viral escape from the host’s immune response, because the region contains B/T-cell epitopes [3–9]. It also has been reported that the preS mutations might influence the serum hepatitis B surface antigen (HBsAg) titer, because the preS region plays a role in HBsAg secretion from hepatocytes [10]. Considering this background, quantification of the preS mutations might improve our understanding of the mechanism of liver disease progression. On the other hand, it is not yet known which preS mutant is most important and how the contribution of the preS mutant to the viral quasispecies affects liver disease progression.

Recently, serum HBsAg quantification became possible and is considered an important viral marker, reflecting intrahepatic hepatitis B virus cccDNA (HBV cccDNA) [11] and, therefore, decreasing or even eliminating serum HBsAg is considered to be and has been proposed as the ultimate goal of anti-HBV therapy. More recently, the serum hepatitis B core-related antigen (HBcrAg) titer, a test developed in Japan to quantify the combined titer of serum hepatitis B core antigen (HBcAg), hepatitis B e antigen (HBeAg) and p22cr antigen (p22crAg) [12, 13], was also reported as an additional marker reflecting intrahepatic HBV cccDNA [14]. Because the presence of preS mutations could affect the serum HBsAg titer, as stated above, the interrelationship among the quasispecies state of preS mutants, HBsAg, HBcrAg and disease advancement is considered rather complicated. However, determining the quantitative interrelationships among these factors might advance our understanding of the pathogenesis of HBV-induced liver disease.

In this study, deep sequencing analysis of preS region was carried out to determine the most relevant preS deletion mutant associated with the development of liver fibrosis in chronic HBV patients and to disclose how the determined preS deletion affects the clinical characteristics, as well as viral markers.

## Results

### Clinical characteristics of the patients

The clinical backgrounds and viral markers of the 90 patients, including 29 inactive carriers, 28 with chronic hepatitis and 33 with cirrhosis, are shown in Table 1.

**Table 1:** Background of the patients.

Factor	Inactive carrier (n = 29)	Chronic hepatitis (n = 28)	Cirrhosis (n = 33)
**Clinical parameters**			
Age (> 65 y/o), n(%)	5 (17%)	2 (8.3%)	9 (27%)
Gender (male)	13 (45%)	14 (50%)	25 (76%)
Platelets (<15 x10^-4^/mm^3^), n(%)	4 (14%)	15 (50%)	28 (85%)
Alb (< 4.0 g/dl), n(%)	2 (6.8%)	6 (20%)	22 (67%)
ALT (>31 IU/l), n(%)	3 (10%)	24 (80%)	20 (61%)
AFP (≥5ng/ml), n(%)	2 (6.8%)	8 (27%)	21 (64%)
FIB-4*>1.45	11 (38%)	20 (71%)	33 (100%)
HCC, n(%)	0 (0%)	1 (3.5%)	14 (42%)
**Viral markers**			
HBeAg (+), n(%)	0 (0%)	10 (33%)	13 (39%)
HBsAg (< 3log IU/ml), n(%)	16 (55%)	12 (42%)	16 (48%)
HBV DNA (>4.0 log IU/ml), n(%)	0 (0%)	16 (53%)	17 (52%)
HBcrAg (log IU/ml), n(%)	5 (17%)	28 (93%)	30 (91%)
Genotype A/B/C, n(%)	1 (3.4%)/ 9 (31%)/ 19 (65.5%)	1 (3.6%)/ 0 (0%)/ 27 (96.4%)	0 (0%)/ 3 (9.1%)/ 30 (90.9%)

Alb, Albumin; ALT, alamine amino transferase; AFP, alfa-fetoprotein; FIB-4, Fibrosis-4; HCC, hepatocellular carcinoma; HBeAg, hepatitis B e antigen; HBsAg, hepatitis B surface antigen; HBV DNA, hepatitis B virus DNA; HBcrAg, hepatitis B core-related antigen.

There were 52 males (58%), and 17% (15/90) were with HCC at the time of enrollment. As to viral markers, 26% (23/90) were HBeAg positive. Median values of HBsAg, HBV DNA, and HBcrAg were 3.0 log IU/ml, 5.5 log IU/ml and 4.2 log IU/ml, respectively. Eighty-four percent (76/90) of the patients were infected with genotype C HBV, while the others were infected with genotype A or B. Male sex, low platelets, low albumin, high alpha fetoprotein (AFP, a tumor marker for HCC), high Fibrosis-4 (FIB-4, an indicator of liver fibrosis) index and HCC history were observed more frequently in the liver cirrhosis group than the inactive carrier and chronic hepatitis groups (Table 1). Moreover, HBeAg positivity, high serum HBV DNA, high HBcrAg and genotype C were also more frequent in the cirrhosis group.

### Mapping of the preS region associated with platelet counts

**Fig 1:**
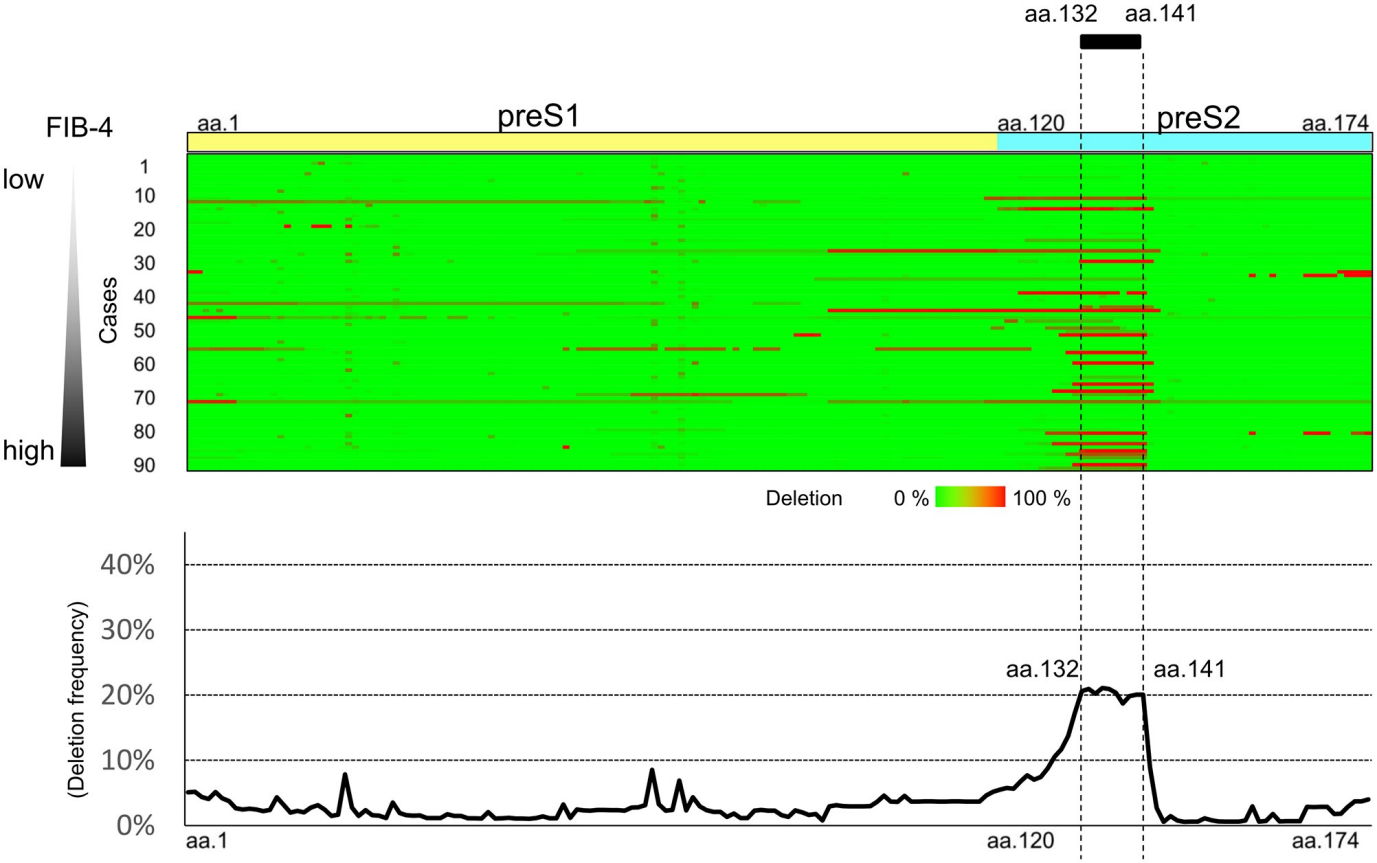
HBV preS deletion mapping using deep sequencing. Deep sequencing of the region of the HBV genome encoding the 174 preS amino acids was carried out and the deleted amino acids were mapped in all 90 patients. Upper panel, the frequency of deletion of each amino acid position in the preS region is expressed for each patient as a color gradient between green and red. In this figure, these sequences are arranged in the order of the FIB-4 values. Lower panel, the average deletion frequency of the preS region per patient is shown.

Deep sequencing of the region of the HBV genome encoding the174 preS amino acids was carried out. For each patient, the presence of amino acid deletions equal to or above the 0.1% cut-off, determined by the control plasmid experiment, was analyzed and the deleted amino acids were mapped for all 90 patients. In Fig 1 (upper panel), the deleted regions are colored red, while the region without deletion is colored green. In this figure, the preS region data of the 90 patients are arranged in the order of FIB-4 values. In Fig 1 (lower panel), the average deletion frequency of preS region per patient is shown. Most of the deletion were in-frame and were without frameshift or stop codon. The region of the genome encoding preS2 codon 132 to 141 was deleted most frequently.

### Deletion frequency in the preS region and its association with clinical factors

**Fig 2:**
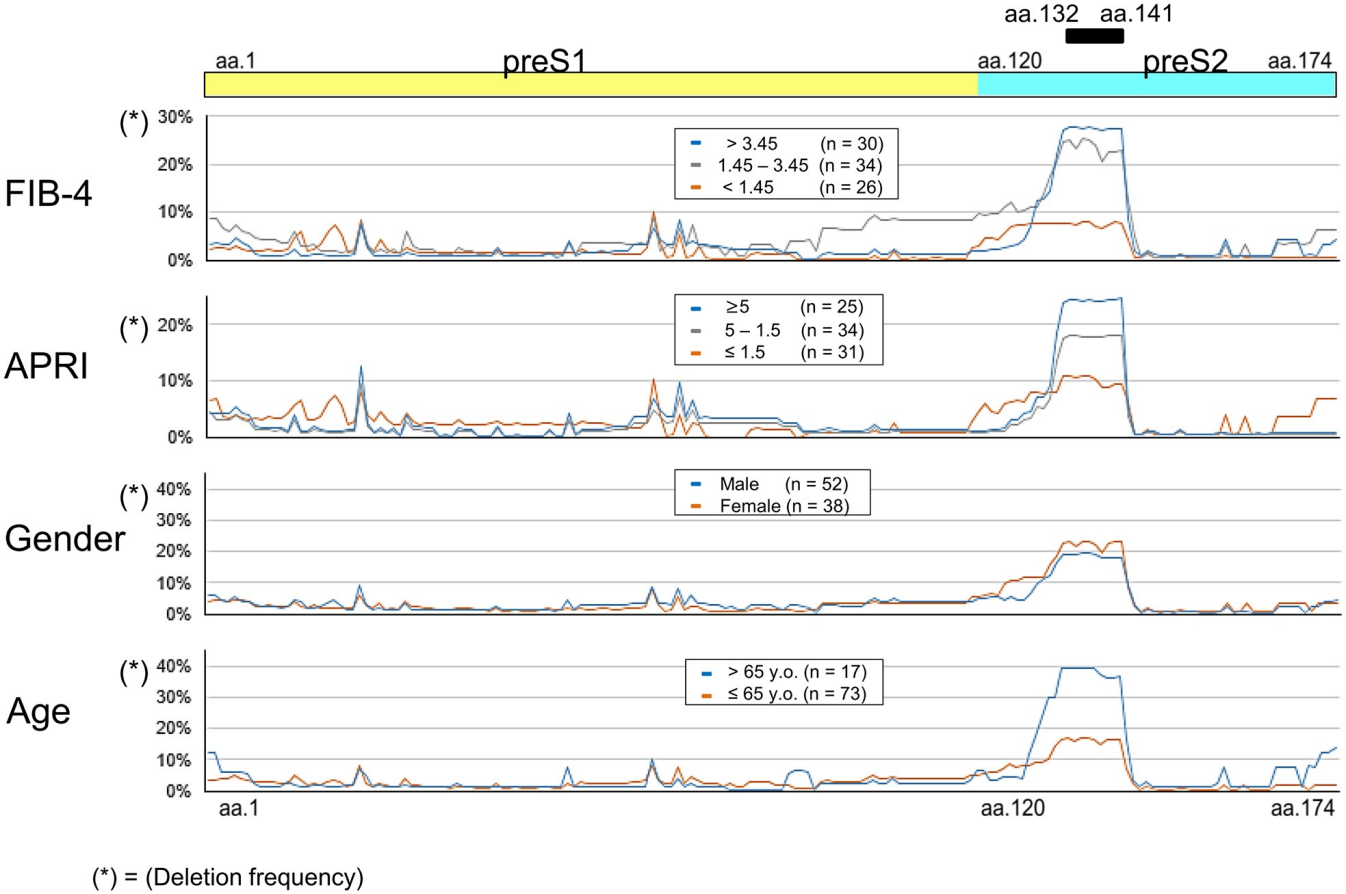
PreS deletion frequency and its association with clinical factors. PreS deletion frequency and its association with gender, age and clinical factors (FIB-4 and APRI). A. The patients were stratified into three groups according to their FIB-4 values (over 3.45; n = 30, 1.45–3.45; n = 34, and < 1.45; n = 26). B. The patients were stratified into three groups (5≤; n = 25, 5–1.5; n = 34, and ≤1.5; n = 31) according to their APRI values. C. The patients were divided into two groups according to their sex (male; n = 52, female; n = 38). D. The patients were stratified into two groups according to their age (> 65 y.o., n = 17, ≤ 65 y.o., n = 73).

The preS deletion frequency and its association with each clinical factors is shown in Fig 2. Based on the FIB-4 values, the patients were stratified into three groups (over 3.45; n = 30, 1.45–3.45; n = 34, and < 1.45; n = 26) and significant differences were observed in aa.132 to aa.141. Specifically, the preS region encoding aa.132 to aa.141 was deleted frequently in patients with a high FIB-4 index. The association of preS deletion frequency with APRI, gender and age was investigated (Fig 2), and it was also found that the preS region encoding aa.132 to aa.141 was deleted frequently in patients with a high aspartate aminotransferase—to—platelet ratio (APRI, and indicator of liver fibrosis) index and those who were elderly, while no association was found with gender.

### Deletion frequency of the preS region and its association with viral markers

**Fig 3:**
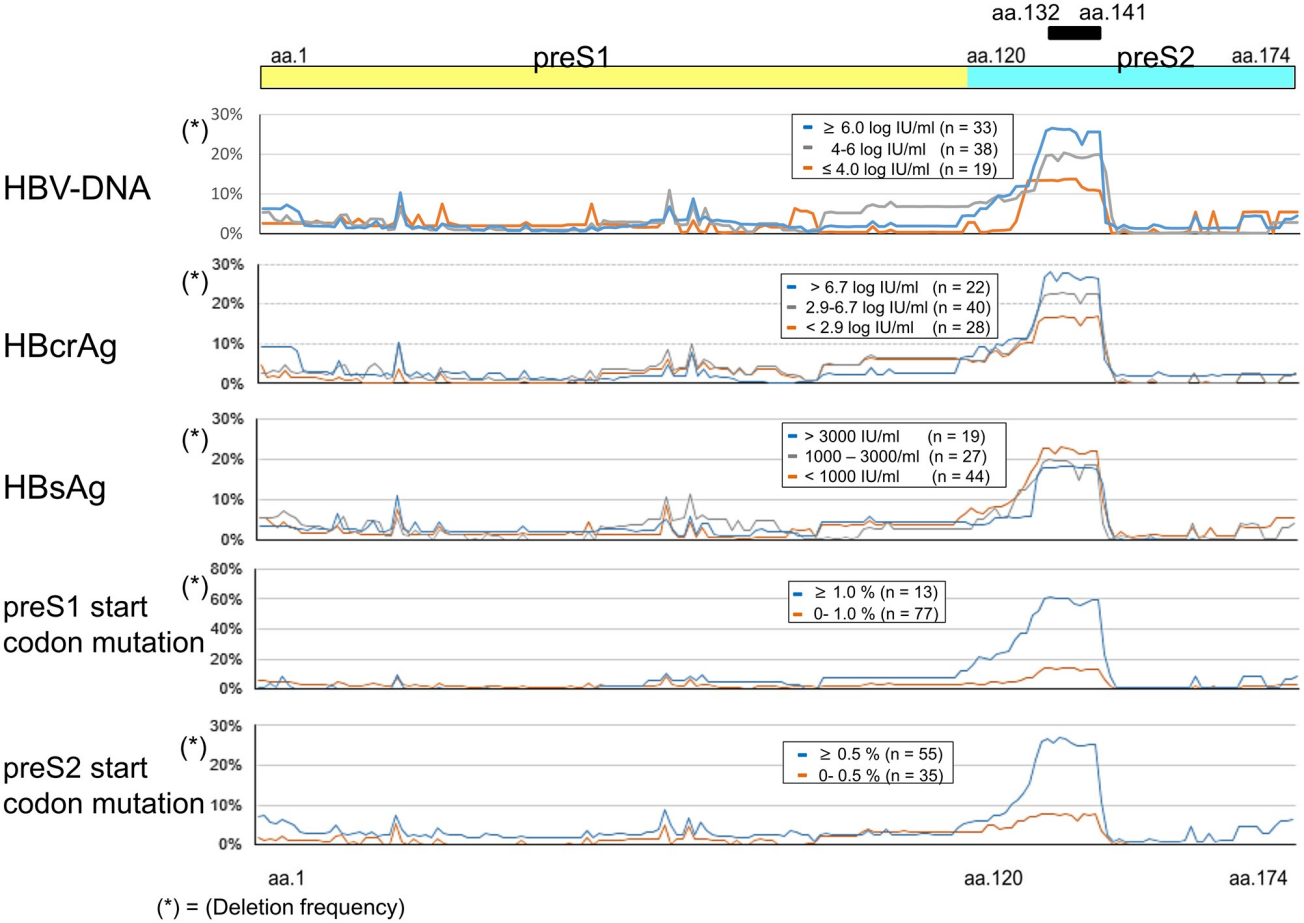
PreS deletion frequency and its association with viral markers. The preS deletion frequency and its association with viral markers (HBV DNA, HBcrAg, HBsAg, preS1 start codon mutation, and preS2 start codon mutation). A, The patients were stratified into three groups according to their HBV DNA titers(≥ 6.0 log IU/ml, n = 33, 4.0–6.0 log IU/ml, n = 38 and ≤ 4.0 log IU/ml, n = 19). B. The patients were stratified into three groups according to their HBcrAg titers (> 6.7 log IU/ml, n = 22, 2.9–6.7 log IU/ml, n = 40 and < 2.9 log IU/ml, n = 28). C. The patients were stratified into three groups according to their HBsAg titers (> 3000 IU/m,; n = 19, 1000–3000 IU/ml, n = 27 and < 1000 IU/ml, n = 44). D. The patients were stratified into two groups according to the frequency of preS1 start codon mutations (≥ 1.0%, n = 13 and 0.0–1.0%, n = 77). E. The patients were stratified into two groups according to the frequency of preS2 start codon mutations (≥ 0.5%, n = 55 and 0.0–0.5%, n = 35).

The preS deletion frequency and its association with viral markers is shown in Fig 3. The preS region encoding aa.132 to aa.141 was deleted frequently in patients with high serum HBV DNA levels, high serum HBcrAg, preS1 start codon mutations and preS2 start codon mutations. Regarding the association with HBsAg, the preS region encoding aa.132 to aa.141 was deleted frequently in those with low HBsAg titers (Fig 3).

### Stratification of patients according to the frequency of preS codon 132-141deletions

Because the data above showed a significant impact of the preS2 codon 132–141 region on clinical, as well as viral markers, we stratified the patients into three groups, according to the frequency of codon 132-141deletions. Group 0–0.1% includes 27 patients with 0% to <0.1% deletions; these patients are considered not to have preS codon 132-141deletions because the deletion rate was below the deep sequencing cut-off. Group 0.1–10% includes 34 patients with ≥0.1% to <10% deletions and Group 10–100% includes 29 patients with ≥10% to 100% deletions. Thus, 63/90 (70%) of the patients had preS codon 132-141deletions to some degree.

**Fig 4:**
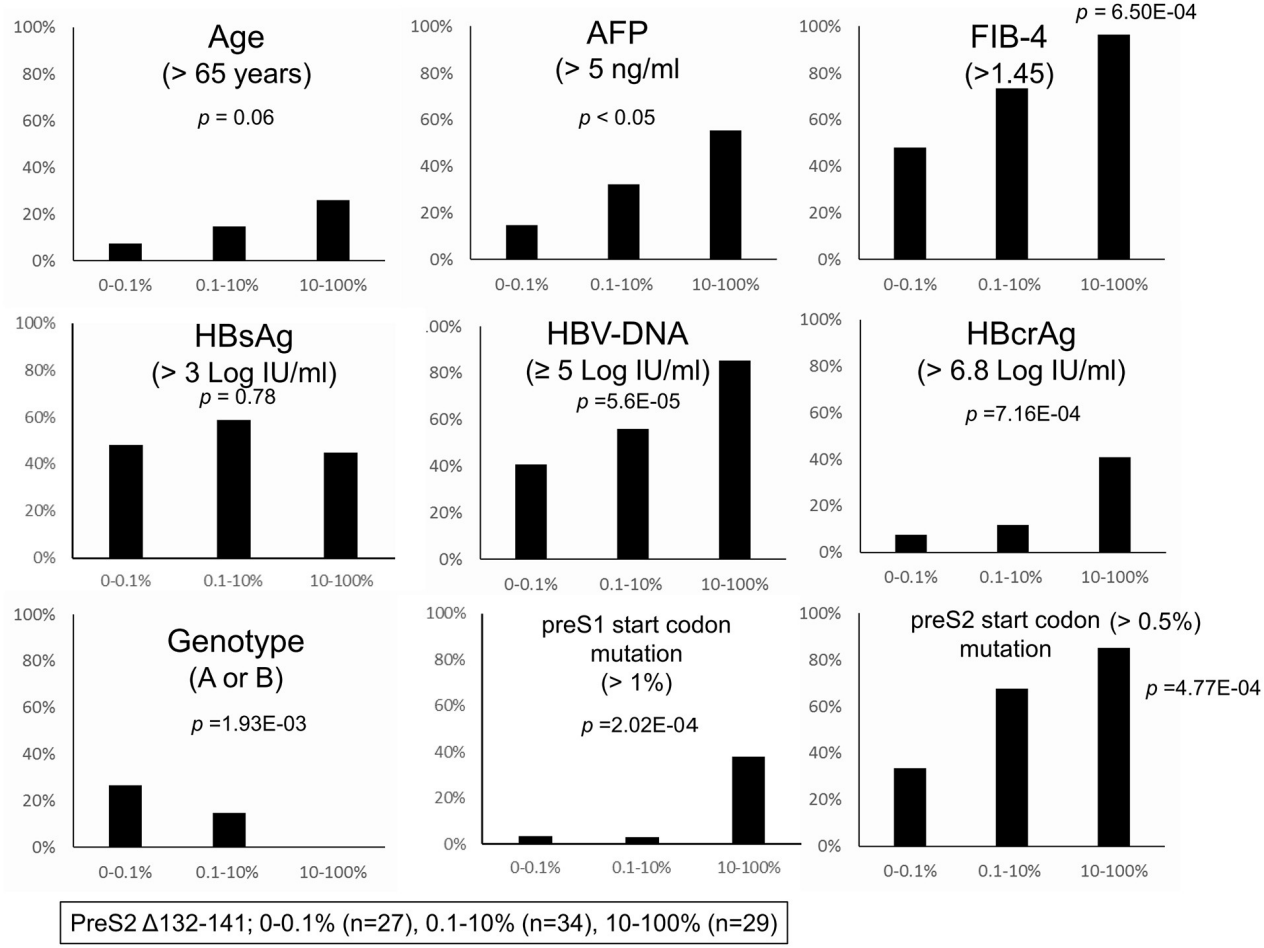
The correlations between the frequency of preS codon 132–141 deletions and host factors and viral markers. The correlations between the frequency of preS codon 132-141deletions deletions and host factors (age, AFP, and FIB-4) and viral markers (HBV DNA, HBcrAg, genotype and preS1/S2 start codon mutations) are demonstrated.

In Fig 4, the correlations between these three groups and clinical factors (age, AFP, and FIB-4) and viral markers (HBV DNA, HBcrAg, genotype, preS1/S2 start codon mutations) were investigated. As shown here, significant and stepwise associations with the preS codon 132-141deletion were observed (Fig 4). It is noteworthy that a very low percentage of preS1/preS2 start codon mutations (> 1% in preS1 and > 0.5% in preS2) was significantly correlated with the preS codon 132-141deletions; this would not have been evident without deep sequencing analysis. Genotypes were also significantly step-wisely associated with preS codon 132-141deletions. While all patients (100%, 29/29) in 10–100% were infected with genotype C, 67% (18/27) in group 0–0.1% patients and 85% (29/34) in group 0.1–10% patients were infected with genotype C.

In S1 Fig a and b, preS deletion map and viral markers in each patient are demonstrated as lists in group 10–100% (a, n = 29) and in group 0–0.1% (b, n = 27).

### Multivariate analysis of independent factors associated with preS codon 132–141 deletions

It was clear that the preS codon 132–141 deletion rate was significantly associated with various factors, therefore, we carried out multivariate analysis to identify factors influenced independently by the deletions (Table 2).

**Table 2:** Univariate and multivariate analysis for factors associated with preS codon 132–141 deletion (≥ 10%).

	Univariate analysis					Multivariate analysis		
	0–10% (n = 61)		10–100% (n = 29)		*p*—value	OR	95% CI	*p*—value
	n	(%)	n	(%)				
Age (> 65 years)	8	(13%)	9	(31%)	< 0.05*	2.1	(0.5–8.6)	0.32
Gender (Male)	37	(61%)	15	(52%)	0.28			
Alb (≤4.0 g/dL)	20	(33%)	10	(34%)	0.66			
ALT (≥ 31 IU/mL)	31	(51%)	16	(55%)	0.44			
AFP (≥ 5 ng/ml)	16	(26%)	15	(52%)	< 0.05*	1.0	(0.3–4.0)	0.95
FIB-4 (> 1.45)	38	(62%)	26	(90%)	< 0.01**	9.0	(1.2–65.6)	< 0.05*
HCC (yes)	10	(16%)	5	(17%)	0.66			
HBsAg (> 3.0 LogIU/mL)	33	(54%)	13	(45%)	0.50			
HBV-DNA (≥ 5.0 LogIU/ml)	30	(49%)	23	(79%)	< 0.01**	0.8	(0.2–3.3)	0.71
HBcrAg (> 6.8 LogIU/ml)	6	(10%)	11	(38%)	< 0.01**	6.8	(1.2–39.5)	< 0.05*
HBeAg (positive)	9	(15%)	14	(48%)	< 0.01**	1.4	(0.3–7.6)	0.66
Genotype (non-C)‡	14	(23%)	0	(0%)	< 0.01**			
preS1 start codon mutation (> 1%)	2	(3%)	11	(38%)	< 0.01**	28.6	(3.5–236.8)	< 0.01**
preS2 start codon mutation (> 0.5%)	32	(52%)	23	(79%)	< 0.01**	1.5	(0.9–2.3)	0.10

Univariate analysis and multivariate were performed with Fisher exact t-test and logistic -regression analysis respectively.

^‡^Genotype was excluded from multivariate analysis because calculation was impossible due to deviated distribution of patients.

* <0.05,

** < 0.01

Multivariate analysis extracted FIB-4, HBcrAg and preS1 start codon mutation as independent factors.

### Impact of preS codon 132–141 deletions on the serum HBsAg and HBcrAg titer, relative to the serum HBV DNA level

**Fig 5:**
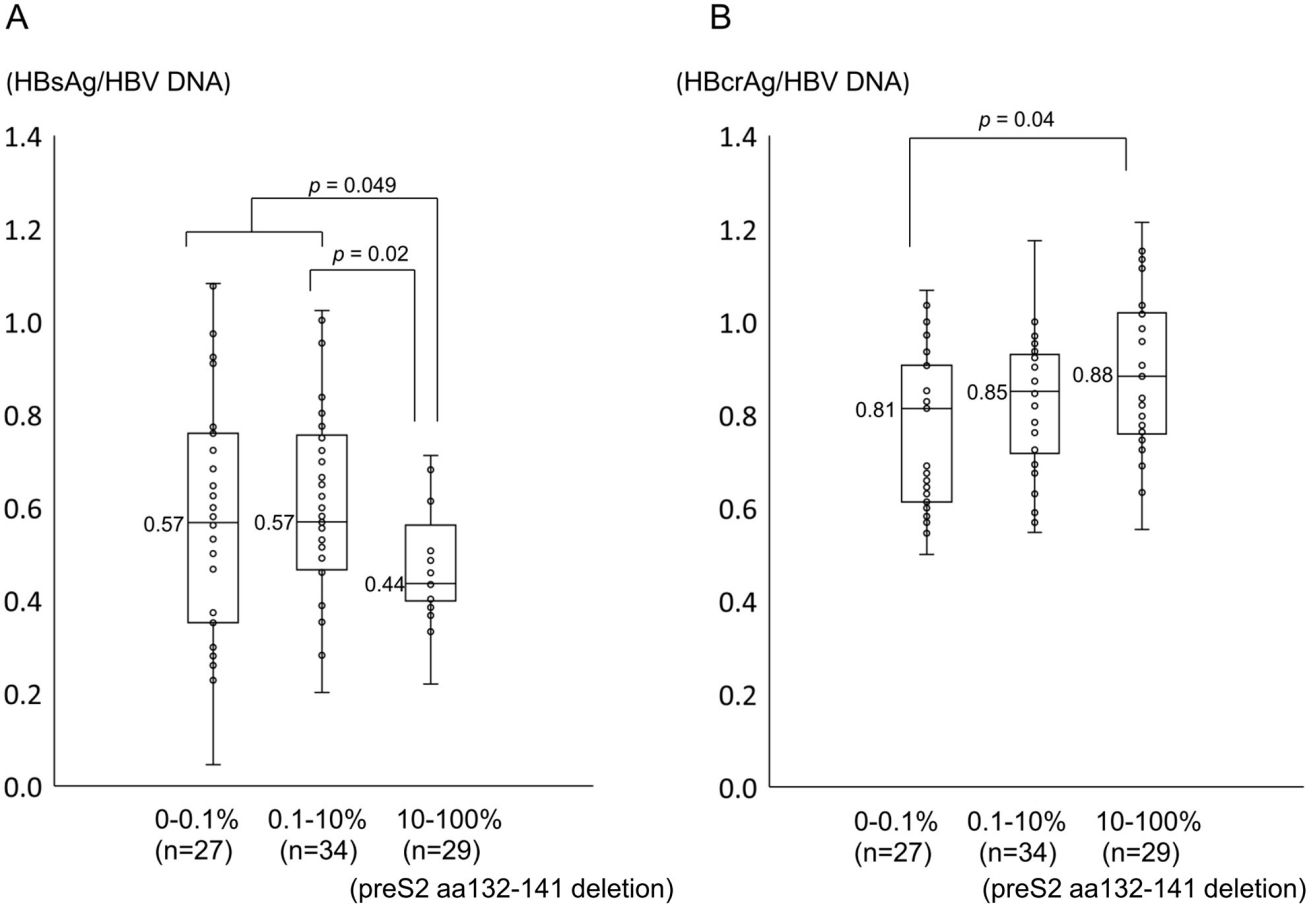
The impact of preS codon 132–141 deletions on the serum HBsAg and HBcrAg titers, relative to the serum HBV DNA level. Left panel, the HBsAg titer relative to the HBV DNA value was significantly lower in cases with high frequencies of mutations (10–100%) than in those with low frequencies. Right panel, the HBcrAg titer relative to the HBV DNA value was significantly higher in cases with high frequencies of mutations (10–100%) compared to those with low frequencies (0–0.1%).

We investigated the influence of preS codon 132–141 deletions on the serum HBV markers, focusing on HBsAg and HBcrAg. Because our interest was to determine how production of these viral antigens from the viral DNA is affected by the presence of preS codon 132–141 deletion, they are expressed as the amount of viral antigen relative to the serum HBV DNA titer: HBsAg/ HBV DNA and HBcrAg/ HBV DNA. As shown in Fig 5, left panel, the HBsAg titer relative to the HBV DNA value was significantly lower in cases with high frequencies (10–100%) of deletions than those with low frequencies. Conversely, the HBcrAg titer relative to the HBV DNA value was significantly higher in cases with high frequencies (10–100%) of deletions than those with low frequencies (0–0.1%, Fig 5, right panel).

### Future NA therapy in patients with preS codon 132–141 deletions

**Fig 6:**
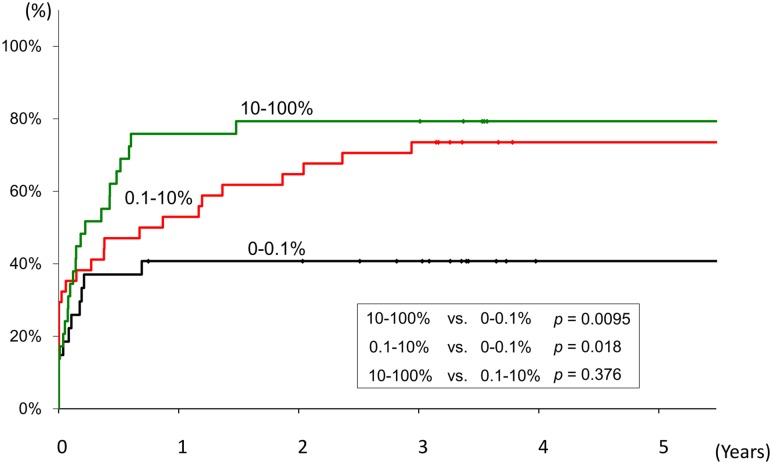
Frequency of the preS codon 132–141 deletions and its influence on the requirement for future nucleoside analogue therapy. Kaplan-Meier curves were drawn according to the frequency of the preS codon 132–141 deletions to determine the influence of the deletions on the requirement for future nucleoside analogue therapy over the ensuing five years. As is shown, compared to the patients with the preS codon 132–141 deletions 0–0.1%, NA therapy was introduced more often in patients with high deletion frequencies (10–100%, p = 0.0095) or with intermediate deletion frequencies (1–10%, p = 0.018).

In order to determine the influence of the preS codon 132–141 deletions on the requirement for future NA therapy, Kaplan-Meier curves were drawn according to the frequency of the preS codon 132–141 deletions. As shown in Fig 6, compared to the patients with the preS codon 132–141 deletions 0–0.1%, NA therapy was introduced more often in patients with high deletion frequencies (10–100%, p = 0.0095) or with intermediate deletion frequencies (1–10%, p = 0.018), over the ensuing five years (Fig 6).

## Discussion

In this study, based on deep sequencing analysis, we have shown that deletions in the preS region of the HBV genome are present in the viral quasispecies of HBV-infected Japanese patients and that deletions of the region encoding codon 132–141 are most frequent. Importantly, the presence of these deletions showed significant positive correlations with fibrosis (FIB-4 and APRI) and high AFP values. Moreover, the presence of these deletions was correlated with viral markers (the HBV DNA titer and HBcrAg titer) and preS1/S2 start codon mutations, even when present as a minor species. From multivariate analysis, advanced fibrosis (FIB-4), high HBcrAg titer, and a high prevalence of preS1 start codon mutations were independently correlated with preS2 codon 132–141 deletions.

It has been reported that preS mutants, including deletion mutants, are found frequently in patients with advanced liver disease, including liver cirrhosis and HCC [6, 7, 15–19] and the mutants have even been found to be correlated with NA resistance [20]. These mutations are considered to be selected as the result of immune pressure, because many T/B-cell epitopes are present in the preS region [6]. On the other hand, although various preS deletion mutants have been reported [6, 7, 10, 21], the most relevant locus in the region affecting clinical characteristics, and the frequency its deletion in individual patients, has been unclear because most of those previous studies, using direct sequencing or sequencing of clones, could not quantify the mutation frequencies. In the real world of chronic viral infection, viruses are considered to infect as a population of different but closely related genomes, the so-called viral quasispecies, and, therefore, preS mutants may be present at various frequencies [22]. In this study, using deep sequencing, we showed that the preS regions also formed a quasispecies in a single patient. By investigating the deletion frequencies, deletion of the region encoding preS codon 132–141 was shown to be the most frequent and common among the patients.

What is the role of preS codon 132–141 deletions in the pathogenesis of HBV-induced liver disease? In previous studies, preS deletions around codon 132–141 were found frequently in patients with liver biopsy specimens with type II ground-glass hepatocytes (GGH) [4, 9, 19, 23]. Because the preS region around codon 132–141 overlaps a CTL epitope, such deletions might occur as a result of immune escape and, therefore, it is plausible that fibrosis is independently associated with codon 132–141 deletions, because advanced fibrosis often is the results of significant CTL-induced inflammation. On the other hand, these deletions may result in the accumulation of HBsAg in the endoplasmic reticulum (ER), when HBsAg secretion is diminished [10, 23]. In such a state, liver damage might be associated with ER stress [24, 25], although cellular damage unrelated to ER stress could also occur [4, 5, 19, 26–28]. Because type-II GGH is often found in advanced liver disease, and is even considered as a precancerous lesion, the results of our study are compatible with those previous studies and rather enhance the correlation of mutations in the viral genome and disease progression.

In this study, it was also demonstrated that the HBV-DNA and HBcrAg titers paralleled the frequency of preS codon 132–141 deletions. Although the mechanism of the correlation remains unclear, it is also plausible that highly replication-competent viruses with high cccDNA transcription activity develop immune-escape mutations more rapidly to escape from hosts’ immune attack. Though high HBV-DNA and HBcrAg titers both reflect high intrahepatic HBV cccDNA titers, as described earlier, HBcrAg might reflect intrahepatic HBV-cccDNA transcription activity more accurately, considering that multivariate analysis showed HBcrAg to be independently associated with the deletions. A recent study also reported the significant association between HBcrAg and intrahepatic HBV-cccDNA transcription activity [29]. On the other hand, HBsAg, another viral marker which also reflects intrahepatic HBV cccDNA [11, 30, 31], was not extracted in this study as a factor associated with the deletions. Though its reasons are not clear, if high HBcrAg titer (or high HBV-DNA titer) reflects robust intrahepatic HBV-cccDNA transcription activity as recently reported [29], HBsAg production per HBV-cccDNA might be even decreased when compared with the production of HBcrAg or HBV-DNA in the presence of preS deletions. In this sense, we re-evaluated HBsAg and HBcrAg titer after correction by HBV-DNA titer (Fig 5). The result of Fig 5a might reflect association among preS deletions, intrahepatic HBsAg accumulation and diminished extracellular HBsAg secretion observed in in-vitro studies [10] while Fig 5b might reflect robust intrahepatic HBV-cccDNA transcription activity of HBcrAg though further studies are needed.

Interestingly, in our study, preS1 start codon mutation was also extracted as an independent variable in its association with the preS2 codon 132–141 deletions, even when present as minor species (> 1%). Although the reason for this is unknown, conservation of the preS1 start codon, which is indispensable for large S antigen production, might not be needed because the large S protein lacking 10 amino acids (codon 132–141) produced in the presence of the preS2 codon 132–141 might have dysfunction. Likewise, conservation of the preS2 start codon, which is indispensable for middle S antigen production might not be needed though its correlation with the deletion might be rather weak. Therefore, we speculate that preS1 (and preS2) start codon mutation might not be a cause but rather a result of the preS2 codon 132–141 deletion, although further studies are needed.

One of the limitations of the study is that we could not obtain liver tissue from all of the patients and, therefore, could not investigate the correlation of serum HBsAg, HBcrAg, HBV DNA and the preS codon 132–141 deletions with intrahepatic HBV and liver histology. Because the ultimate goal of anti-HBV therapy is the elimination of HBV from the liver, the association of those markers with liver histology, HBV cccDNA titer and the integration of viral DNA into chromosomal DNA would reveal further the status of HBV-induced liver disease.

In conclusion, quasispecies analysis of preS deletions in the HBV genome using deep sequencing revealed a close correlation between these deletions and the state of liver disease, as well as HBV markers, even when they are present as minor populations. Understanding the interrelationship among those viral markers in association with the state of liver disease would further advance our understanding of the mechanisms of liver disease progression.

## Patients and methods

### Patients

Ninety patients chronically infected with HBV and followed-up at Yamanashi University Hospital after 2004 were included in the study. In order to include patients with advanced diseases as well as inactive carriers, 61 consecutive patients clinically diagnosed as cirrhosis or chronic hepatitis and 29 consecutive patients clinically diagnosed as inactive carriers were enrolled. All patients also met the following criteria: (1) Had not received NA therapy previously at the time of enrollment. (2) Serum was available for HBV sequence analysis. (3) Were hepatitis C antibody negative. (4) Had no other forms of hepatitis, such as primary biliary cirrhosis, autoimmune liver disease or alcoholic liver disease. (5) Were free of co-infection with human immunodeficiency virus. All enrolled patients were positive for HBsAg. HBV DNA was measured by the Quantiplex HBV DNA assay (Bayer Diagnostics, Emeryville, CA, USA), transcription-mediated amplification assay (Chugai Diagnostics Science Co., Ltd., Tokyo, Japan), or COBAS Amplicor HBV Monitor Test v2.0 (Roche Diagnostics, Indianapolis, IN, USA). The clinical characteristics of these patients are shown in Table 1.

We firstly intended to include larger number of patients in this study. However, at that time, NA therapy was introduced in Japan, and NA treatment was started for most active hepatitis (chronic hepatitis and liver cirrhosis) patients. Therefore, it became difficult to include more patients, especially patients with active disease.

### Ethics statement

Informed consent was obtained for participation in the study protocol, which had been approved by the Human Ethics Review Committee of Yamanashi University. All included patients were adult. Written informed consent or ethics committee approved opt-out consent was obtained from all individual patients included in the study.

### DNA extraction, PCR, and deep sequencing

The preS region of HBV DNA was amplified from the patients’ sera using two-step PCR. The first and the second-round primers are shown in the S1 Table. Briefly, viral DNA was extracted from stored sera using QIAamp MinElute Virus Spin Kits (QIAGEN, Tokyo, Japan) with QIAcube (QIAGEN), and then the extracted DNA was subjected to two-step PCR. The primers for the second-round PCR had bar codes 10 nucleotides in length attached and these differed for each sample, so that the PCR products from each sample were identifiable (S1 Table). After the PCR products were quantified using a Pico Green dsDNA Assay Kit (Invitrogen, Tokyo, Japan), the concentrations of the samples were adjusted to a common value and the samples were pooled.

Libraries were then subjected to emulsion PCR, the enriched DNA beads were loaded onto a picotiter plate and pyrosequencing was carried out with a Roche GS Junior/454 sequencing system using titanium chemistry (Roche, Branford, CT). The Roche Variant Analyzer version 2.5pl (Roche) and Microsoft Excel (Microsoft, Tokyo, Japan) were used for the analysis. The method for pyrosequencing was described previously in more detail [32].

### Detection of mutations and deletions in preS1 and preS2 regions using deep sequencing

Deep sequencing involved pyro-sequencing of the 522 nt PCR amplified preS1/S2 region. The average depth was approximately 2279 reads and the proportion of preS1 and preS2 start codon mutations and preS1and preS2 deletions was determined for each patient. The cut-off for the presence of mutations was set at 0.1%, based on a control experiment using the plasmid template.

### Statistical analysis

Statistical differences in the parameters, including all available demographic, biochemical, hematological, and virological variables, were determined for the various patient groups by Fisher’s exact probability test for categorical variables. The odds ratios and 95% confidence intervals were calculated. The Cochran-Armitage trend test was used to look for trends in the categorical data among three groups, divided on the basis of differences in the preS deletion frequency. A logistic regression analysis was used to investigate independent variables associated with the preS codon 132–141 deletion. In order to evaluate the requirement for future NA treatment, Kaplan-Meier curves were drawn and the log-rank test was performed. P values of <0.05 by the two-tailed test were considered to indicate statistical significance.

## Supporting information

S1 Table The first and the second-round primers for the preS hepatitis B virus genomic region. The primers for the second-round PCR had bar codes 10 nucleotides in length attached and these differed for each sample, so that the PCR products from each sample were identifiable.
(XLSX)

S1 Fig preS deletion map and viral markers in group 10–100% and group 0–0.1%. In S1 Fig, preS deletion map and viral markers in each patient are demonstrated as lists in group 10–100% (1a, n = 29) and in group 0–0.1% (1b, n = 27).
(PDF)
