# Supplementary material for: HBV preS deletion mapping using deep sequencing demonstrates a unique association with viral markers
Source: PLoS One. 2019 Feb 22;14(2):e0212559. doi: 10.1371/journal.pone.0212559 (PMC6386350; doi:10.1371/journal.pone.0212559)
Supplement: S1 Fig — In S1 Fig, preS deletion map and viral markers in each patient are demonstrated as lists in group 10–100% (1a, n = 29) and in group 0–0.1% (1b, n = 27). (PDF) [file pone.0212559.s002.pdf]

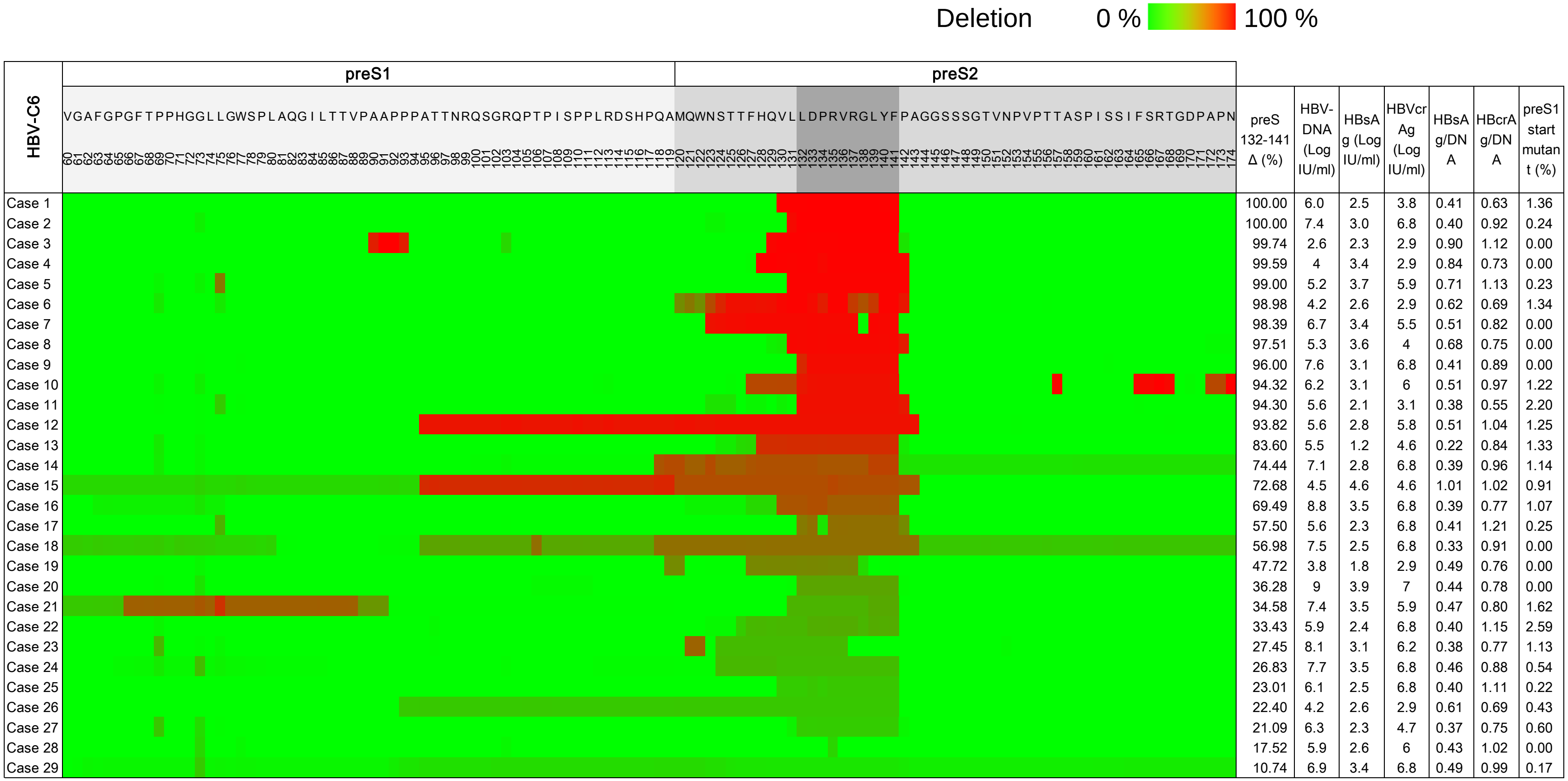

Case	preS 132-141 Δ (%)	HBV-DNA (Log IU/ml)	HBsAg (Log IU/ml)	HBVcrAg (Log IU/ml)	HBsAg/DNA	HBcrAg/DNA	preS1 start mutant (%)
Case 1	100.00	6.0	2.5	3.8	0.41	0.63	1.36
Case 2	100.00	7.4	3.0	6.8	0.40	0.92	0.24
Case 3	99.74	2.6	2.3	2.9	0.90	1.12	0.00
Case 4	99.59	4	3.4	2.9	0.84	0.73	0.00
Case 5	99.00	5.2	3.7	5.9	0.71	1.13	0.23
Case 6	98.98	4.2	2.6	2.9	0.62	0.69	1.34
Case 7	98.39	6.7	3.4	5.5	0.51	0.82	0.00
Case 8	97.51	5.3	3.6	4	0.68	0.75	0.00
Case 9	96.00	7.6	3.1	6.8	0.41	0.89	0.00
Case 10	94.32	6.2	3.1	6	0.51	0.97	1.22
Case 11	94.30	5.6	2.1	3.1	0.38	0.55	2.20
Case 12	93.82	5.6	2.8	5.8	0.51	1.04	1.25
Case 13	83.60	5.5	1.2	4.6	0.22	0.84	1.33
Case 14	74.44	7.1	2.8	6.8	0.39	0.96	1.14
Case 15	72.68	4.5	4.6	4.6	1.01	1.02	0.91
Case 16	69.49	8.8	3.5	6.8	0.39	0.77	1.07
Case 17	57.50	5.6	2.3	6.8	0.41	1.21	0.25
Case 18	56.98	7.5	2.5	6.8	0.33	0.91	0.00
Case 19	47.72	3.8	1.8	2.9	0.49	0.76	0.00
Case 20	36.28	9	3.9	7	0.44	0.78	0.00
Case 21	34.58	7.4	3.5	5.9	0.47	0.80	1.62
Case 22	33.43	5.9	2.4	6.8	0.40	1.15	2.59
Case 23	27.45	8.1	3.1	6.2	0.38	0.77	1.13
Case 24	26.83	7.7	3.5	6.8	0.46	0.88	0.54
Case 25	23.01	6.1	2.5	6.8	0.40	1.11	0.22
Case 26	22.40	4.2	2.6	2.9	0.61	0.69	0.43
Case 27	21.09	6.3	2.3	4.7	0.37	0.75	0.60
Case 28	17.52	5.9	2.6	6	0.43	1.02	0.00
Case 29	10.74	6.9	3.4	6.8	0.49	0.99	0.17

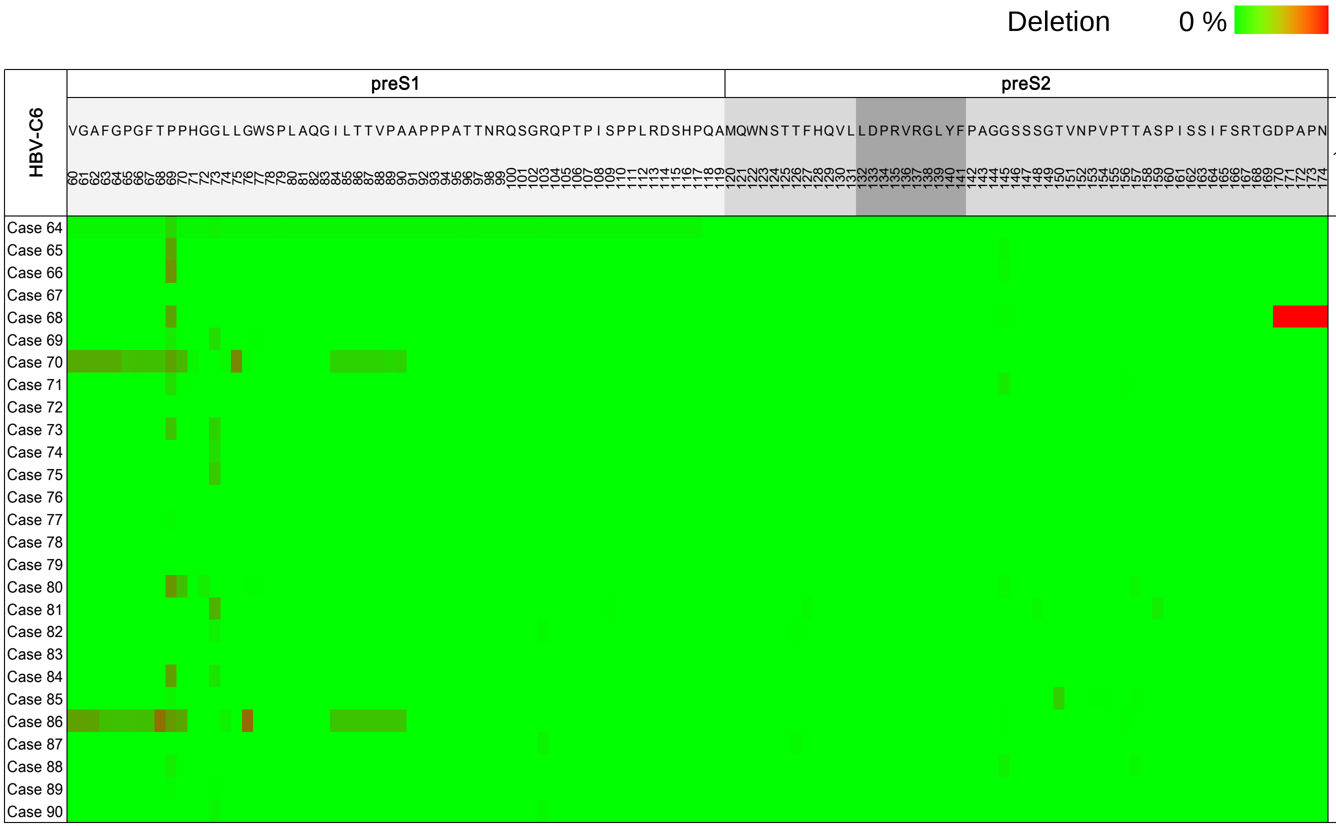

	preS 132-141 Δ (%)	HBV-DNA (Log IU/ml)	HBsAg (Log IU/ml)	HBVcrAg (Log IU/ml)	HBsAg/DNA	HBcrAg/DNA	preS1 start mutant (%)
Case 64	0.00	7.7	2.9	5.1	0.37	0.66	0.31
Case 65	0.00	3.2	0.1	2.9	0.05	0.91	0.00
Case 66	0.00	4.4	1.0	2.9	0.23	0.66	0.00
Case 67	0.00	3.5	1.1	2.9	0.31	0.83	0.00
Case 68	0.00	4.2	1.2	2.9	0.28	0.69	0.00
Case 69	0.00	5.9	1.8	6.3	0.30	1.07	0.08
Case 70	0.00	5.1	1.8	2.9	0.36	0.57	0.00
Case 71	0.00	7.5	1.9	4.5	0.26	0.60	0.20
Case 72	0.00	3.1	2.0	2.9	0.65	0.94	0.00
Case 73	0.00	4.4	2.3	3.6	0.53	0.82	0.00
Case 74	0.00	6.7	2.4	3.9	0.35	0.58	0.00
Case 75	0.00	3.5	2.7	2.9	0.76	0.83	0.18
Case 76	0.00	4.6	2.8	2.9	0.60	0.63	17.50
Case 77	0.00	5.8	2.9	2.9	0.50	0.50	0.00
Case 78	0.00	2.8	3.0	2.9	1.08	1.04	0.00
Case 79	0.00	4	3.1	4	0.77	1.00	0.00
Case 80	0.00	4.3	3.1	2.9	0.72	0.67	0.39
Case 81	0.00	5.5	3.2	3	0.58	0.55	0.00
Case 82	0.00	3.5	3.2	5.8	0.92	1.66	0.35
Case 83	0.00	4.8	3.3	2.9	0.68	0.60	0.00
Case 84	0.00	5.4	3.4	4.6	0.62	0.85	0.10
Case 85	0.00	6.2	3.5	3.8	0.57	0.61	0.16
Case 86	0.00	3.5	3.8	3.4	1.08	0.97	0.18
Case 87	0.00	8.2	3.8	6.8	0.47	0.83	0.00
Case 88	0.00	4.5	4.1	2.9	0.91	0.64	0.42
Case 89	0.00	4.3	4.2	3.5	0.97	0.81	0.17
Case 90	0.00	7.5	4.2	6.8	0.56	0.91	0.15
